# Outcomes in high and low volume hospitals in patients with acute hematochezia in a cohort study

**DOI:** 10.1038/s41598-021-99832-6

**Published:** 2021-10-13

**Authors:** Naoki Ishii, Naoyoshi Nagata, Katsumasa Kobayashi, Atsushi Yamauchi, Atsuo Yamada, Jun Omori, Takashi Ikeya, Taiki Aoyama, Naoyuki Tominaga, Yoshinori Sato, Takaaki Kishino, Tsunaki Sawada, Masaki Murata, Akinari Takao, Kazuhiro Mizukami, Ken Kinjo, Shunji Fujimori, Takahiro Uotani, Minoru Fujita, Hiroki Sato, Sho Suzuki, Toshiaki Narasaka, Junnosuke Hayasaka, Tomohiro Funabiki, Yuzuru Kinjo, Akira Mizuki, Shu Kiyotoki, Tatsuya Mikami, Ryosuke Gushima, Hiroyuki Fujii, Yuta Fuyuno, Naohiko Gunji, Yosuke Toya, Kazuyuki Narimatsu, Noriaki Manabe, Koji Nagaike, Tetsu Kinjo, Yorinobu Sumida, Sadahiro Funakoshi, Kana Kawagishi, Tamotsu Matsuhashi, Yuga Komaki, Kuniko Miki, Kazuhiro Watanabe, Fumio Omata, Yasutoshi Shiratori, Noriatsu Imamura, Takahiko Yano, Mitsuru Kaise

**Affiliations:** 1 Division of Gastroenterology, Tokyo Shinagawa Hospital, 6-3-22 Higashi-Ooi, Shinagawa-ku, Tokyo, 140-8522 Japan; 2 Department of Gastroenterological Endoscopy, Tokyo Medical University, Tokyo, Japan; 3 Department of Gastroenterology and Hepatology, National Center for Global Health and Medicine, Tokyo, Japan; 4 Department of Gastroenterology, Tokyo Metropolitan Bokutoh Hospital, Tokyo, Japan; 5 Department of Gastroenterology and Hepatology, Kitano Hospital, Tazuke Kofukai Medical Research Institute, Osaka, Japan; 6 Department of Gastroenterology, Graduate School of Medicine, The University of Tokyo, Tokyo, Japan; 7 Department of Gastroenterology, Nippon Medical School, Graduate School of Medicine, Tokyo, Japan; 8 Department of Gastroenterology, St. Luke’s International University, Tokyo, Japan; 9 Department of Gastroenterology, Hiroshima City Asa Citizens Hospital, Hiroshima, Japan; 10 Department of Gastroenterology, Saga Medical Center Koseikan, Saga, Japan; 11 Division of Gastroenterology and Hepatology, Department of Internal Medicine, St Marianna University School of Medicine, Kanagawa, Japan; 12 Department of Gastroenterology and Hepatology, Center for Digestive and Liver Diseases, Nara City Hospital, Nara, Japan; 13 Department of Endoscopy, Nagoya University Hospital, Aichi, Japan; 14 Department of Gastroenterology, National Hospital Organization Kyoto Medical Center, Kyoto, Japan; 15 Department of Gastroenterology, Tokyo Metropolitan Cancer and Infectious Diseases Center Komagome Hospital, Tokyo, Japan; 16 Department of Gastroenterology, Oita University, Oita, Japan; 17 Department of Gastroenterology, Fukuoka University Chikushi Hospital, Fukuoka, Japan; 18 Department of Gastroenterology, Chiba Hokusoh Hospital, Nippon Medical School, Chiba, Japan; 19 Department of Gastroenterology, Japanese Red Cross Shizuoka Hospital, Shizuoka, Japan; 20 Division of Endoscopy and Ultrasonography, Department of Clinical Pathology and Laboratory Medicine, Kawasaki Medical School General Medical Center, Okayama, Japan; 21 Division of Gastroenterology, Graduate School of Medical and Dental Sciences, Niigata University, Niigata, Japan; 22 Department of Gastroenterology and Hepatology, Center for Digestive Disease and Division of Endoscopy, University of Miyazaki Hospital, Miyazaki, Japan; 23 Department of Gastroenterology, University of Tsukuba, Ibaraki, Japan; 24 Division of Endoscopic Center, University of Tsukuba Hospital, Ibaraki, Japan; 25 Department of Gastroenterology, Toranomon Hospital, Tokyo, Japan; 26 Department of Emergency Medicine, Fujita Health University Hospital, Aichi, Japan; 27 Emergency and Critical Care Center, Saiseikai Yokohamashi Tobu Hospital, Kanagawa, Japan; 28 Department of Gastroenterology, Naha City Hospital, Okinawa, Japan; 29 Department of Internal Medicine, Tokyo Saiseikai Central Hospital, Tokyo, Japan; 30 Department of Gastroenterology, Shuto General Hospital, Yamaguchi, Japan; 31 Division of Endoscopy, Hirosaki University Hospital, Aomori, Japan; 32 Department of Gastroenterology and Hepatology, Graduate School of Medical Sciences, Kumamoto University, Kumamoto, Japan; 33 Department of Gastroenterology and Hepatology, National Hospital Organization Fukuokahigashi Medical Center, Fukuoka, Japan; 34 Department of Medicine and Clinical Science, Graduate School of Medical Sciences, Kyushu University, Fukuoka, Japan; 35 Department of Gastroenterology, Fukushima Medical University, Fukushima, Japan; 36 Division of Gastroenterology, Department of Internal Medicine, Iwate Medical University, Iwate, Japan; 37 Department of Internal Medicine, National Defense Medical College, Saitama, Japan; 38 Division of Endoscopy and Ultrasonography, Department of Clinical Pathology and Laboratory Medicine, Kawasaki Medical School, Okayama, Japan; 39 Department of Gastroenterology and Hepatology, Suita Municipal Hospital, Osaka, Japan; 40 Department of Endoscopy, University of the Ryukyus Hospital, Okinawa, Japan; 41 Department of Gastroenterology, National Hospital Organization Kyushu Medical Center, Fukuoka, Japan; 42 Department of Gastroenterological Endoscopy, Fukuoka University Hospital, Fukuoka, Japan; 43 Department of Gastroenterology, School of Medicine, Kitasato University, Kanagawa, Japan; 44 Department of Gastroenterology and Neurology, Akita University Graduate School of Medicine, Akita, Japan; 45 Digestive and Lifestyle Diseases, Kagoshima University Graduate School of Medical and Dental Sciences, Kagoshima, Japan

**Keywords:** Gastroenterology, Colonoscopy, Gastrointestinal diseases

## Abstract

Outcomes of acute lower gastrointestinal bleeding have not been compared according to hospital capacity. We aimed to perform a propensity score-matched cohort study with path and mediation analyses for acute hematochezia patients. Hospitals were divided into high- versus low-volume hospitals for emergency medical services. Rebleeding and death within 30 days were compared. Computed tomography, early colonoscopy (colonoscopy performed within 24 h), and endoscopic therapies were included as mediators. A total of 2644 matched pairs were yielded. The rebleeding rate within 30 days was not significant between high- and low-volume hospitals (16% vs. 17%, *P* = 0.44). The mortality rate within 30 days was significantly higher in the high-volume cohort than in the low-volume cohort (1.7% vs. 0.8%, *P* = 0.003). Treatment at high-volume hospitals was not a significant factor for rebleeding (odds ratio [OR] = 0.91; 95% confidence interval [CI], 0.79–1.06; *P* = 0.23), but was significant for death within 30 days (OR = 2.03; 95% CI, 1.17–3.52; *P* = 0.012) on multivariate logistic regression after adjusting for patients’ characteristics. Mediation effects were not observed, except for rebleeding within 30 days in high-volume hospitals through early colonoscopy. However, the direct effect of high-volume hospitals on rebleeding was not significant. High-volume hospitals did not improve the outcomes of acute hematochezia patients.

## Introduction

Medical centers are classified as high or low-volume centers in terms of the treatment strategies followed and clinical outcomes achieved for different diseases^1–3^. The outcomes following the management of acute pancreatitis and upper gastrointestinal bleeding, as well as the outcomes of high-risk surgeries, were noted to be superior in high-volume centers^1–3^. However, the differences between these settings in terms of clinical outcomes for the management of patients with acute hematochezia—specifically acute lower gastrointestinal bleeding (ALGIB)—have not been sufficiently investigated.

The incidence of ALGIB, including colonic diverticular bleeding, has been increasing in recent years^4^. The global coronavirus disease (COVID-19) pandemic has strained health systems worldwide and created a need to use evidence-based strategies to effectively prioritize the use of limited medical resources shared between patients with COVID-19 and other urgent conditions, including ALGIB^5^. Role allotments, especially for emergency diseases, are required according to hospital characteristics. If outcomes of ALGIB are better in high-volume hospitals than in low-volume hospitals, ALGIB cases should be primarily managed at high-volume hospitals with sufficient medical resources. On the other hand, because the clinical course of most ALGIB cases, including colonic diverticular hemorrhage, are generically mild with spontaneous resolution of bleeding episodes in 70–80% of cases^6,7^, there may be a possibility of equal outcomes irrespective of hospital capacity.

Recently, path analysis and a generalized structural equation model (GSEM) have been used not only in causal pathways but also for the evaluation of complex network and mediation analysis^8,9^. As acute hematochezia has many possible causes and the diagnosis of ALGIB requires computed tomography (CT) and colonoscopy, management strategies remain complicated despite the currently available guidelines^10–14^. The introduction of path and mediation analyses may be required for the evaluation of these diagnostic and treatment modalities for patients with ALGIB.

This propensity score-matched cohort study aimed to compare outcomes and management strategies for patients with acute hematochezia treated at the high-volume and low-volume hospitals for emergency medical services and perform path and mediation analyses that influence clinical outcomes using GSEM.

## Results

### Patient characteristics

**Figure 1:**
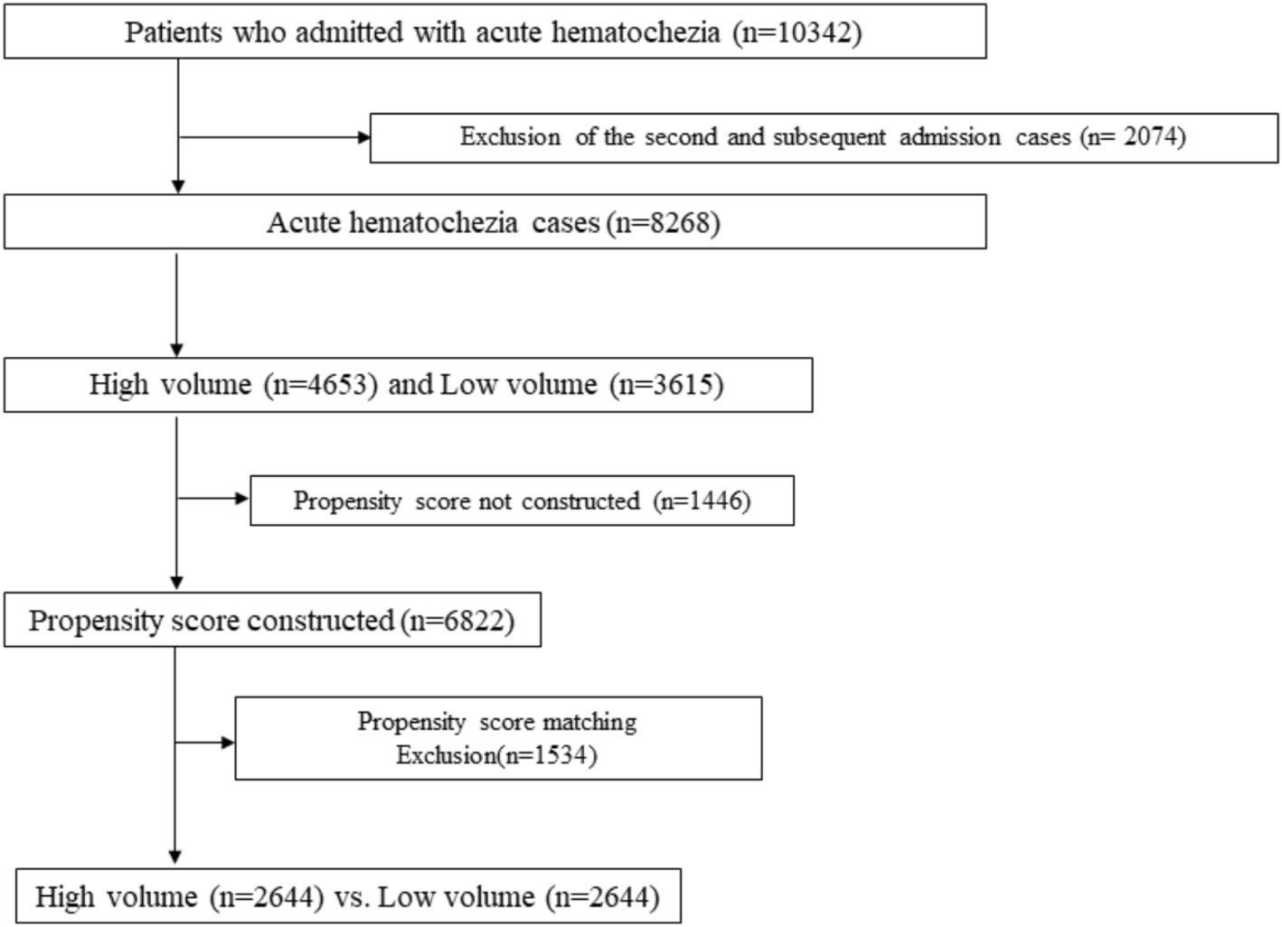
Study flow diagram of this study. *Note* A total of 10,343 patients were admitted due to acute hematochezia. Because one of the important outcomes was death within 30 days, the second and subsequent admission events were excluded to reduce selection bias, and the first admission cases were included in the study. A total of 8268 cases were divided into high- and low-volume groups. Propensity scores were constructed using complete data cases. A total of 2644 matched pairs were yielded.

The area under the receiver operating curve of propensity scores for high-volume settings was 0.57 (95% confidence interval [CI], 0.56–0.59). Propensity scores were constructed in 6822 complete-data cases and a total of 2644 matched pairs were yielded (Fig. 1). The characteristics of unmatched and matched patients in the high- and low-volume hospitals are shown in Table 1. The absolute value of standardized differences determined after balance checking was less than 0.1 for all variables.

**Table 1:** Characteristics of patients with acute hematochezia in unmatched and matched cohorts: high-volume versus low-volume hospitals.

	Unmatched cohort (n = 8268)			Matched cohort (n = 5288)			
	High volume hospitals (n = 4653)	Low-volume hospitals (n = 3615)	*P* value	High-volume hospitals (n = 2644)	Low-volume hospitals (n = 2644)	ASD	*P* value
Age, mean (SD)	70 (15)	71 (14)	0.0001	72 (14)	71 (14)	0.086	0.002
Male, n (%)	2784 (60)	2200 (61)	0.35	1673 (63)	1618 (61)	0.043	0.12
PH colonic surgery, n (%)	315 (6.8)	279 (7.6)	0.1	203 (7.7)	198 (7.5)	0.007	0.80
PH CDB, n (%)	639 (14)	612 (17)	< 0.0001	501 (19)	436 (16)	0.064	0.019
Diabetes mellitus, n (%)	868 (19)	644 (18)	0.33	486 (18)	491 (19)	0.005	0.86
Hypertension, n (%)	2488 (56)	1980 (55)	0.24	1467 (55)	1454 (55)	0.010	0.72
Dyslipidemia, n (%)	1270 (27)	863 (24)	< 0.0001	596 (23)	655 (25)	0.053	0.056
CCI≧2, n (%)	1736 (37)	1288 (36)	0.12	946 (36)	962 (36)	0.013	0.65
NSAIDs, n (%)	518 (11)	427 (12)	0.34	339 (13)	320 (12)	0.022	0.43
Anticoagulants, n (%)	565 (12)	513 (14)	0.006	431 (16)	389 (15)	0.044	0.11
Antiplatelets, n (%)	1284 (28)	1004 (28)	0.86	770 (29)	757 (29)	0.011	0.69
PS3, n (%)	212 (4.6)	271 (7.5)	< 0.0001	199 (7.5)	173 (6.5)	0.038	0.16
SBP≦100, n (%)	651 (14)	471 (13)	0.17	368 (14)	379 (14)	0.023	0.40
PR ≧100, n (%)	965 (21)	676 (19)	0.017	482 (18)	524 (20)	0.040	0.14
Unconsciousness, n (%)	299 (6.4)	287 (8.0)	0.008	257 (9.7)	222 (8.4)	0.046	0.094
Hemoglobin, g/dl (SD)	11.2 (2.7)	11.1 (2.6)	0.21	10.8 (2.7)	11.0 (2.6)	0.054	0.051
Platelet, 10^4^/μl (SD)	22.1 (8.4)	21.6 (8.6)	0.014	21.1 (7.3)	21.6 (8.5)	0.054	0.049
Albumin, g/dl (SD)	3.7 (0.7)	3.7 (0.7)	0.03	3.6 (0.7)	3.6 (0.6)	0.052	0.06
PT-INR (SD)	1.16 (0.84)	1.15 (0.59)	0.52	1.14 (0.60)	1.15 (0.59)	0.010	0.71

A two-tailed *P*-value < 0.05 was considered to indicate statistical significance.

*ASD* absolute standardized difference, *SD* standard deviation, *PH* past history, *CDB* colonic diverticular bleeding, *NSAIDs* non-steroidal anti-inflammatory drugs, *CCI* Charlson comorbidity index, *PS* performance status, *SBP* systolic blood pressure, *PR* pulse rate, *PT-INR* prothrombin-time international normalized ratio.

### Diagnosis of ALGIB

The performances of CT and colonoscopy are shown in Table 2. CT and enhanced CT were performed more frequently in high-volume hospitals than in low-volume hospitals (80% vs. 67%, *P* < 0.0001 and 76% vs. 67%, *P* < 0.0001, respectively). However, the proportions of colonoscopy and early colonoscopy performed were significantly lower in the high-volume hospitals than in low-volume hospitals (85% vs. 95%, *P* < 0.0001 and 55% vs. 72%, *P* < 0.0001, respectively). CT was more frequently used as the first-line diagnostic modality in high-volume hospitals than in low-volume hospitals (77% vs. 61%, *P* < 0.0001).

**Table 2:** Management and outcomes in patients with acute hematochezia in the unmatched and the matched cohorts: high-volume versus low-volume hospitals.

	Unmatched cohort (n = 8268)			Matched cohort (n = 5288)		
	High-volume hospitals (n = 4653)	Low-volume hospitals (n = 3615)	*P* value	High volume hospitals (n = 2644)	Low-volume hospitals (n = 2644)	*P* value
CT, n (%)	3595 (77)	2224 (62)	< 0.0001	2113 (80)	1751 (67)	< 0.0001
CT as a first-diagnostic method, n (%)	3458 (75)	2019 (56)	< 0.0001	2041 (77)	1605 (61)	< 0.0001
Enhanced CT out of total CT cases, n (%)	2735 (76)	1509 (68)	< 0.0001	1614 (76)	1180 (67)	< 0.0001
Colonoscopy, n (%)	3898 (84)	3384 (94)	< 0.0001	2237 (85)	2517 (95)	< 0.0001
Colonoscopy as a first-diagnostic method, n (%)	1003 (22)	1471 (41)	< 0.0001	506 (19)	951 (36)	< 0.0001
Early colonoscopy, n (%)	2157 (55)	2495 (74)	< 0.0001	1234 (55)	1807 (72)	< 0.0001
Endoscopic therapy, n (%)	1083 (23)	1106 (31)	< 0.0001	633 (24)	788 (30)	< 0.0001
Indirect clip, n (%)	458 (9.8)	537 (15)	< 0.0001	236 (8.9)	372 (14)	< 0.0001
Direct clip, n (%)	267 (5.7)	151 (4.2)	0.001	162 (6.1)	102 (3.9)	< 0.0001
EBL, n (%)	214 (4.6)	261 (7.22)	< 0.0001	150 (5.7)	197 (7.5)	0.009
EDSL, n (%)	71 (1.5)	8 (0.2)	< 0.0002	42 (1.6)	6 (0.2)	< 0.0001
IVR, n (%)	57 (1.2)	49 (1.4)	0.6	35 (1.3)	45 (1.7)	0.26
Surgery, n (%)	59 (1.3)	30 (0.8)	0.055	34 (1.3)	25 (1)	0.24
Rebleeding within 30 days, n (%)	692 (15)	609 (17)	0.014	436 (16)	457 (17)	0.44
Death within 30 days, n (%)	66 (1.4)	31 (0.9)	0.019	45 (1.7)	21 (0.8)	0.003
Transfused RPBCs, packs, median (IQR)	0 (0–2)	0 (0–2)	< 0.0001	0 (0–4)	0 (0–2)	< 0.0001
Length of hospital stay, days, median (IQR)	7 (5–11)	7 (5–11)	0.016	7 (5–12)	7 (5–11)	0.25

A two-tailed *P*-value < 0.05 was considered to indicate statistical significance.

*CT* computed tomography, *EBL* endoscopic band ligation, *EDSL* Endoscopic detachable snare ligation, *IVR* interventional radiology, *PRBCs* packed red blood cells, *IQR* interquartile range.

The sources of bleeding are listed in Table 3. Although diverticular bleeding was the most common cause of acute hematochezia in both groups, the proportion of definitive diverticular bleeding was significantly lower in the high-volume hospitals than in low-volume hospitals (19% vs. 26%, *P* < 0.0001). The proportion of patients with upper gastrointestinal bleeding was significantly higher in the high-volume hospitals than in low-volume hospitals (3.4% vs. 0.3%, *P* < 0.0001).

**Table 3:** Diagnosis in patients with acute hematochezia in the unmatched and the matched cohorts: high-volume versus low-volume hospitals.

	Unmatched cohort (n = 8268)			Matched cohort (n = 5288)		
	High-volume hospitals (n = 4653)	Low-volume hospitals (n = 3615)	*P* value	High-volume hospitals (n = 2644)	Low-volume hospitals (n = 2644)	*P* value
Definitive DB, n (%)	821 (18)	920 (25)	< 0.0001	510 (19)	697 (26)	< 0.0001
Presumptive, n (%)	1632 (35)	1474 (41)	< 0.0001	1035 (39)	1105 (42)	0.05
Ischemic colitis, n (%)	615 (13)	251 (6.9)	< 0.0001	279 (11)	160 (6.1)	< 0.0001
Colorectal cancer, n (%)	109 (2.3)	54 (1.5)	0.006	57 (2.2)	49 (1.9)	0.43
Metastatic cancer, n (%)	11 (0.2)	5 (0.1)	0.31	7 (0.3)	5 (0.2)	0.56
Other cancer, n (%)	8 (0.2)	0 (0)	0.013	5 (0.2)	0 (0)	0.025
Polyp, n (%)	18 (0.4)	18 (0.5)	0.45	10 (0.4)	16 (0.6)	0.24
Infectious colitis, n (%)	106 (2.3)	23 (0.6)	< 0.0001	37 (1.4)	11 (0.4)	< 0.0001
IBD, n (%)	101 (2.2)	86 (2.4)	0.53	29 (1.1)	57 (2.2)	0.002
Post-endoscopic therapy, n (%)	232 (5.0)	214 (5.9)	0.062	91 (3.4)	113 (4.3)	0.12
Post-colectomy, n (%)	6 (0.13)	8 (0.22)	0.31	4 (0.15)	6 (0.23)	0.53
Drug-induced ulcer, n (%)	4 (0.09)	5 (0.14)	0.47	2 (0.08)	5 (0.2)	0.27
Non-specific ulcer, n (%)	34 (0.7)	16 (0.3)	0.094	24 (0.9)	13 (0.5)	0.07
Non-specific colitis, n (%)	31 (0.7)	8 (0.2)	0.003	18 (0.7)	7 (0.3)	0.027
Dieulafoy's ulcer, n (%)	6 (0.1)	5 (0.1)	0.91	3 (0.11)	5 (0.2)	0.48
Diverticulitis, n (%)	6 (0.13)	1 (0.03)	0.12	1 (0.04)	1 (0.04)	1
Varices, n (%)	11 (0.2)	7 (0.2)	0.68	9 (0.3)	6 (0.2)	0.44
Radiation proctocolitis, n (%)	27 (0.6)	27 (0.8)	0.35	16 (0.6)	17 (0.6)	0.86
Rectal ulcer, n (%)	135 (2.9)	109 (3.0)	0.76	87 (3.3)	86 (3.3)	0.94
Vascular ectasia, n (%)	52 (1.1)	64 (1.8)	0.012	32 (1.2)	53 (2.0)	0.022
Hemorrhoids, n (%)	101 (2.2)	72 (2.0)	0.57	51 (1.9)	52 (1.8)	0.92
Anal diseases, n (%)	4 (0.09)	6 (0.17)	0.3	3 (0.11)	4 (0.15)	0.71
Upper GI bleeding, n (%)	140 (3.0)	8 (0.2)	< 0.0001	87 (3.4)	7 (0.3)	< 0.0001
Small intestinal bleeding, n (%)	113 (2.4)	83 (2.3)	0.69	65 (2.5)	60 (2.3)	0.65
Others, n (%)	23 (0.5)	9 (0.3)	0.075	14 (0.5)	6 (0.2)	0.73
Not identified, n (%)	307 (6.6)	142 (3.9)	< 0.0001	168 (6.4)	103 (3.9)	< 0.0001

A two-tailed *P*-value < 0.05 was considered to indicate statistical significance.

*DB* diverticular bleeding, *IBD* inflammatory bowel disease, *GI* gastrointestinal.

### Treatments and outcomes

Treatments and outcomes in the high- and low-volume hospitals are shown in Table 2. Endoscopic therapy was performed less frequently in high-volume hospitals than in low-volume hospitals (24% vs. 30%, *P* < 0.0001). The rebleeding rate within 30 days was not significantly different between the two cohorts (16% vs. 17%, *P* = 0.44). The number of deaths within 30 days was significantly higher in the high-volume cohort than in the low-volume cohort (45 [1.7%] vs. 21 [0.8%], *P* = 0.003). A significant difference was observed regarding transfusion of packed red blood cells between the high- and low-volume hospitals (0 [interquartile range (IQR), 0–4] vs. 0 [IQR, 0–2], *P* < 0.0001). No significant difference was found in the length of stay between the two groups (7 [IQR, 5–12] vs. 7 [IQR, 5–11], *P* = 0.25).

Being treated at a high-volume hospital was not a significant factor for rebleeding within 30 days, but was a significant factor for death within 30 days according to the multivariate logistic regression data after adjusting for patients’ characteristics in the matched cohort, respectively (odds ratio [OR], 0.91, 95% CI, 0.79–1.06, *P* = 0.23; OR, 2.03; 95% CI, 1.17–3.52; *P* = 0.012) (Table 4). Being treated at a high-volume hospital was a significant factor in increasing the amount of packed red blood cells transfused and the length of stay on the multivariate linear regression after controlling for patients’ characteristics (Table 4). Statistical difference was also observed in the unmatched cohort.

**Table 4:** Association with rebleeding and death within 30 days, the amount of PRBCs, and length of hospital stay in the unmatched and the matched cohorts.

Unmatched cohort (n = 6822)		Matched cohort (n = 5288)	
**Rebleeding within 30 days**			
*High-volume*			
OR, 95% CI^a^	*P* value^a^	OR, 95% CI^a^	*P* value^a^
0.89, 0.78–1.02	0.091	0.91, 0.79–1.06	0.23
**Death within 30 days**			
*High-volume*			
OR, 95% CI^a^	*P* value^a^	OR, 95% CI^a^	*P* value^a^
1.96, 1.18–3.25	0.010	2.03, 1.17–3.52	0.012
**Transfused RPBCs, packs**			
*High-volume*			
β coefficient, 95% CI^a^	*P* value^a^	β coefficient, 95% CI^a^	*P* value^a^
0.48, 0.28–0.68	< 0.0001	0.54, 0.31–0.78	< 0.0001
**Length of hospital stay, days**			
*High-volume*			
β coefficient, 95% CI^a^	*P* value^a^	β coefficient, 95% CI^a^	*P* value^a^
1.08, 0.54–1.63	< 0.0001	0.99, 0.41–1.57	0.001

^a^Adjusted for patients’ characteristics used for the construction of propensity scores.

A two-tailed *P*-value < 0.05 was considered to indicate statistical significance.

*OR* odds ratio, *CI* confidence interval, *PRBCs* packed red blood cells.

#### Path and mediation analyses using a GSEM

The results of path analyses between hospital characteristics and rebleeding and death within 30 days with or without mediators using a GSEM are demonstrated in Table 5 and Supplementary Note. CT was used as a first-line diagnostic modality, early colonoscopy, and endoscopic therapies were included as the mediators. Considering coefficient differences between the results with and without the use of mediators, absolute coefficient differences were larger on death within 30 days.

**Table 5:** Association of hospital characteristics with rebleeding and death within 30 days by using generalized structural equation model (GSEM).

Association without mediators			Association with mediators		
High-volume hospitals	Coefficient, 95%CI	*P*-value	High-volume hospitals	Coefficient, 95%CI^a^	*P*-value^a^
Rebleeding within 30 days < High-volume hospitals	− 0.057, − 0.201–0.087	0.441	Rebleeding within 30 days < High-volume hospitals	0.099, − 0.054–0.252	0.203
Death within 30 days < High-volume hospitals	0.771, 0.251–1.292	0.004	Death within 30 days < High-volume hospitals	0.423, − 0.169–1.014	0.161

^a^Computed tomography used as a first-line diagnostic modality, early colonoscopy, and endoscopic therapies were included as the mediators in the generalized structural equation model (GSEM). The differences between coefficients with and without mediators were larger on death within 30 days. A two-tailed P-value < 0.05 was considered to indicate statistical significance.

*CI* confidence interval.

The results of mediation analyses in each mediator are demonstrated in Table 6. Indirect effects were not statistically significant in all analyses, except for early colonoscopy on the association between high-volume hospitals and rebleeding within 30 days (coefficient, − 0.083, 95% CI, − 0.114–0.053: *P* < 0.0001). However, the direct effect, the subtraction of the indirect effect from the total effect, was not statistically significant on the association.

**Table 6:** Mediation analysis between hospital characteristics and rebleeding and death within 30 days in the matched cohort.

Independent variable, high-volume hospitals					
Dependent variable, rebleeding within 30 days			Dependent variable, death within 30 days		
Mediator, CT first	Coefficient, 95% CI	*P*-value	Mediator, CT first	Coefficient, 95% CI	*P*-value
Total effect	− 0.057, − 0.201–0.088	0.443	Total effect	0.771, 0.266–1.277	0.003
Indirect effect	− 0.002, − 0.028–0.024	0.872	Indirect effect	0.020, − 0.086–0.125	0.715
Direct effect	− 0.054, − 0.203–0.094	0.472	Direct effect	0.752, 0.232–1.272	0.005

Mediator, early colonoscopy	Coefficient, 95% CI	*P*-value	Mediator, early colonoscopy	Coefficient, 95% CI	*P*-value
Total effect	0.030, -0.115–0.175	0.684	Total effect	0.459, − 0.301–1.219	0.236
Indirect effect	− 0.083, − 0.114–0.052	< 0.0001	Indirect effect	0.028, − 0.088–0.143	0.638
Direct effect	0.113, − 0.030–0.257	0.122	Direct effect	0.431, -0.293–1.155	0.243

Mediator, endoscopic therapies	Coefficient, 95% CI	*P*-value	Mediator, endoscopic therapies	Coefficient, 95% CI	*P*-value
Total effect	− 0.057, − 0.204–0.091	0.451	Total effect	0.771, 0.162–1.381	0.013
Indirect effect	− 0.004, − 0.013–0.006	0.430	Indirect effect	0.014, − 0.016–0.045	0.357
Direct effect	− 0.053, − 0.201–0.096	0.486	Direct effect	0.757, 0.141–1.373	0.016

Computed tomography used as a first-line diagnostic modality, early colonoscopy, and endoscopic therapies were included in the mediation analysis as the mediators. A two-tailed *P*-value < 0.05 was considered to indicate statistical significance.

*CT* computed tomography, *CI* confidence interval.

## Discussion

To the best of our knowledge, this study is the first to compare the management and clinical outcomes of patients with acute hematochezia according to hospital characteristics and perform path and mediation analyses. Unlike in the study for upper gastrointestinal bleeding^1^, high-volume hospitals for emergency medical services did not improve rebleeding and death within 30 days compared to low-volume hospitals in the present study, although adjunctive outcomes such as performance of CT and colonoscopy, and the bleeding causes were significantly different between the two groups. Patients with acute hematochezia could have equal treatment outcomes regardless of the hospital capacity if CT and emergency endoscopy were available 24/7.

Although CT is more commonly performed in high-volume hospitals, the use of colonoscopy is the reverse of CT. Upper gastrointestinal bleeding should be suspected in cases of gastrointestinal bleeding with unstable vitals, even for patients presenting with acute hematochezia. CT was recommended as a first-line diagnostic modality for these patients^10–14^. Our finding that CT was performed more frequently in high-volume hospitals might explain the high proportion of patients with unstable vital signs, such as patients with upper gastrointestinal bleeding who presented with acute hematochezia. The proportions of early colonoscopy and endoscopic therapies were lower in high-volume hospitals than in low-volume hospitals in the present study. The systematic review and meta-analysis demonstrated that early colonoscopy was associated with the performance of endoscopic therapies^15^. In the mediation analyses shown in Table 6, the indirect effect of early colonoscopy was significant, showing an inverse effect on rebleeding within 30 days in high-volume hospitals. The lower performance of early colonoscopy, a negative coefficient in this path, and the association of early colonoscopy with rebleeding within 30 days, a positive coefficient, contributed to this result (Table 2 and Supplementary Note) because the indirect effects were calculated by multiplication of each coefficient on a path. However, further studies are required to clarify the influence of early colonoscopy by comparing early vs. non-early colonoscopy groups.

The most common cause of acute hematochezia was diverticular bleeding (Table 3). However, the proportion of definitive diverticular bleeding was significantly lower in high-volume hospitals. The performance of CT as the first-line diagnostic modality and early colonoscopy seemed to be conflicting in the diagnosis proportion of definitive diverticular bleeding because the more introduction of CT or early colonoscopy could have the possibility of increasing the definitive diagnosis^16,17^. The role of CT as the first-line diagnostic should be elucidated after time.

The higher performance of hemostatic modalities used was quite different (Table 2). The difference in the diagnosis of acute hematochezia might have influenced the selection of treatment modalities. A few recent studies have reported the therapeutic utility of ligation therapy for colonic diverticular bleeding^18,19^. Moreover, the guidelines for endoscopic clipping recommend that clips be placed onto the vessels by grasping directly, not in a zipper fashion, to ensure hemostatic effects^10–14^. However, the reason for the significant difference in the treatment methods between the groups was not clear in the present study.

No significant difference was found in terms of the rates of rebleeding. Although the differences in causes and treatment modalities for ALGIB could reasonably be expected to impact the rebleeding rates, the lack of a significant between-group difference in terms of this parameter is noteworthy when planning the management of ALGIB. On the other hand, death within 30 days, the amount of packed red blood cells transfused, and length of stay were less favorable in high-volume hospitals. The differences in bleeding causes might contribute to these results, wherein a higher proportion of neoplastic lesions and upper gastrointestinal bleeding was seen in high-volume hospitals (Table 3). These results were quite different from the previous studies regarding upper gastrointestinal bleeding, acute pancreatitis, and high-risk surgeries in terms of better outcomes in high-volume hospitals^1–3^. We were not able to identify and include other confounders to influence these results in the present study.

We performed path and mediation analyses using propensity score-matched data and included candidate mediators in a colonoscopy-based management strategy (Supplementary Note). The coefficient difference between the results with or without the use of mediators composed of CT, early colonoscopy, and endoscopic therapies had a larger effect for high-volume hospitals on death within 30 days (Table 5). The indirect effect of early colonoscopy contributed to rebleeding within 30 days in high-volume hospitals possibly due to the lower performance of early colonoscopy as described before. However, the direct effect of high-volume hospitals on rebleeding was not significant as the total effect. Although the selected diagnostic and treatment modalities contributed to death within 30 days considering the coefficients differences in the path analysis (Table 5), the indirect effects on death within 30 days, one of the most important outcomes, were not significant through these mediators (Table 6). The diagnostic and treatment modalities may not substantially influence the associations between hospital characteristics and outcomes in the colonoscopy-based management of patients with acute hematochezia. These findings are intriguing and warrant further investigation.

The present study had some limitations. First, as a retrospective cohort study, the possibility of selection bias could not be eliminated. Moreover, although the accuracy of the collected data was verified multiple times, the risk of misclassification cannot be ruled out. Second, although measured confounders could be balanced using propensity score matching, unmeasured confounders could not be balanced in the present study. In addition, other variables might be required to be included for elucidating the association between hospital types and outcomes. Third, we performed path and mediation analyses on a colonoscopy-based strategy. However, because there are different paths, such as interventional radiology for the management of patients with acute hematochezia^10–14^, additional path and mediation analyses were required to evaluate the mediation effects between hospital characteristics and outcomes.

This study has several strengths. First, there was a higher proportion of definitive diagnosis of acute hematochezia, which was made based on colonoscopy and/or CT (unknown bleeding etiology 5.2% in the CODE BLUEJ-Study vs. 22.8% in the United Kingdom Study), and important outcomes were evaluated using a database with a robust proportion of definitive diagnoses^20,21^. Second, this study is the first to report a comparison of management and clinical outcomes for patients with acute hematochezia according to hospital characteristics and perform path and mediation analyses. Several guidelines for the management of ALGIB have been published^10–14^. However, there is little information on the association between hospital characteristics and clinical outcomes, types of hospitals that should receive patients with acute hematochezia, and mediation effects on important outcomes. Therefore, the results of this study could be helpful to guide patients with acute hematochezia and emergency transport services to an appropriate center and construct management strategies for patients with acute hematochezia.

Mediation effects were not observed, except for rebleeding within 30 days in high-volume hospitals through early colonoscopy. High-volume hospitals for emergency medical services did not improve the outcomes of patients with acute hematochezia. This suggests that patients with ALGIB have equal treatment outcomes regardless of the hospital capacity to manage the condition.

## Methods

### Patients and database development

We performed a retrospective cohort study using a propensity score drawing from a national large-scale database of ALGIB in Japan, the Colonic Diverticular Bleeding Leaders Update Evidence from the Multicenter Japanese Study (CODE BLUE J-Study)^20^. Forty-nine hospitals in Japan collaborated to build a database of patients aged > 20 years hospitalized with acute hematochezia—regardless of presentation with diarrhea, abdominal pain, or fever—between January 2010 and December 2019. The characteristics of the 49 hospitals are listed in Supplementary Table 1. Patient characteristics were collected from medical charts and endoscopy databases at each hospital. The characteristics included age, sex, height, body weight, Eastern Cooperative Oncology Group Performance Status (PS)^22^, vital signs (systolic blood pressure and heart rate), laboratory data, comorbidities, use of non-steroidal anti-inflammatory drugs (NSAIDs), results and timing of enhanced or plain CT, colonoscopy, endoscopic therapies, and clinical outcomes including rebleeding, thromboembolic events, and deaths. Collected data were checked by the research center. Three to four confirmation processes to ensure accurate data collection and correct fit for the required structures were required and performed between the research center and each hospital to minimize misclassifications and develop a robust database from the medical charts and endoscopy database in each hospital. Active ascertainment of patients’ data by use of telephone or mailing was not performed. The details of the CODE BLUE J-Study are described in a previous report^20^.

### Hospital characteristics and outcome measures

All 49 hospitals were equipped to perform CT and emergency endoscopy which were available 24/7. The diagnosis of patients with acute hematochezia, mainly ALGIB, was based on the colonoscopy and/or CT findings, mainly referring to previous reports^16,17^. Diverticular bleeding was divided into definitive diverticular bleeding with stigmata of recent hemorrhage (SRH; active bleeding, non-bleeding visible vessel, or adherent clot) and presumptive diverticular bleeding without SRH and with no other bleeding sources by colonoscopy^23^. On enhanced CT, diverticula with or without extravasation were considered definitive or presumptive bleeding sources, respectively. If bleeding sources were not identified by colonoscopy and/or CT, then capsule endoscopy, balloon endoscopy, or esophagogastroduodenoscopy were performed, as appropriate, to reevaluate the bleeding sources. The origin of acute hematochezia was categorized as “unknown” for patients in whom bleeding sources could not be definitively diagnosed.

The primary outcomes were rebleeding and mortality within 30 days. Massive rectal bleeding after interventions, such as colonoscopy, interventional arterial embolization, and surgery, and decreased hemoglobin levels, were considered as rebleeding. The amount of transfused packed red blood cells and length of stay were secondary outcomes. Bleeding causes, management strategies such as performance of CT, enhanced CT, colonoscopy, and early colonoscopy (performed within 24 h after admission), and treatment methods were also collected and assessed as adjunctive outcomes.

### Study setting

This study set is demonstrated in Fig. 1. As death within 30 days was one of the important outcomes, the second and subsequent admission events were excluded to reduce selection bias, and the first admission cases were included in the study. A total of 8268 cases were divided into high- and low-volume groups.

We obtained information about the number of emergency medical services from the gastroenterologists participating in the present study. The cut-off value was the 70 percentiles of 49 hospitals. High-volume emergency medical service was defined as > 5000 services provided in the 2019 calendar year (15 hospitals). Low volume was defined for < 5000 cases in that same time interval (34 hospitals).

### Statistical analysis

A logistic regression model was used to calculate the case propensity score based on age (years), sex, history of colonic diverticular bleeding and colectomy, presence of diabetes mellitus, hypertension, and dyslipidemia; Charlson Comorbidity Index (equal to or more than 2)^24^; use of NSAIDs, anticoagulants, and antiplatelets; equal to or more than performance status 3; vital signs (systolic blood pressure ≤ 100 mmHg, heart rate ≥ 100/minute) at the initial visit; and laboratory data at admission (hemoglobin, platelet, serum albumin, and prothrombin time-international normalized ratio)^25^. One-to-one matching was performed between the two groups using the nearest neighbor method with a caliper width of 0.2 of the standard deviation of the logit of the propensity score.

Continuous and categorical variables of patient characteristics were compared using the student’s t-test and a chi-square test, respectively. The amount of transfused packed red blood cells and length of stay was compared using the Mann–Whitney U test. Multivariate logistic regression was used to examine the effects of hospital characteristics on rebleeding and deaths within 30 days while controlling for patient demographics. Because the amount of packed red blood cells and that of the length of stay were compared as continuous variables between high- and low-volume hospitals, these outcomes were evaluated using multivariate linear regressions while controlling for patient demographics. Multivariate logistic and linear regression were required in the matched cohort for the double robustness of evaluating the effect of hospital volume in addition to the propensity score-matched analyses. In addition, because there can be the risk of losing a substantial number of patients in the propensity score-matched analyses, multivariate regression was also used in the unmatched cohort.

At last, because colonoscopy was considered a mainstay for the management of ALGIB^10–14^, path analysis was performed mainly based on colonoscopy-based strategy by GSEM, and mediation effect was analyzed between hospital characteristics and rebleeding and death within 30 days. As CT, the timing of colonoscopy, and endoscopic therapies were considered candidate mediators which may influence outcomes^15–17,23^, these factors were included in the path. A Stata command, Idecomp, was used for the mediation analysis^26^. A two-tailed *P*-value < 0.05 was considered to indicate statistical significance. All analyses were performed using STATA version 16 (Stata Corp, College Station, TX, USA; https://www.stata.com).

This study was performed in accordance with the 1964 Declaration of Helsinki and its later amendments or comparable ethical standards. The need to obtain patient informed consent was waived by the central institution (Tokyo Medical University) because of the retrospective nature of the study. The central institution (Tokyo Medical University) has a licensing committee/Institutional review board to approve the study on human participants. The study protocol was approved by the Institutional Ethics Committee of Tokyo Medical University (T2019-0244). A single IRB review was applied to this study and approved in all hospitals (Supplementary Table 1).

## Supplementary Information

Supplementary Information 1.

Supplementary Information 2.

## Data Availability

If requested, access to the data of this study can be reviewed through the principal investigator of this study and the corresponding author, although this data is not available to the public due to privacy and ethical restrictions.

## Abbreviations

ALGIB: Acute lower gastrointestinal bleeding
CDB: Colonic diverticular bleeding
CCI: Charlson comorbidity index
CODE BLUE J-Study: Colonic Diverticular Bleeding Leaders Update Evidence from the Multicenter Japanese Study
COVID-19: Coronavirus disease 2019
CRP: C-reactive protein
CT: Computer tomography
EBL: Endoscopic band ligation
EDSL: Endoscopic detachable snare ligation
IQR: Interquartile range
IVR: Interventional radiology
LOS: Length of hospital stay
NSAIDs: Non-steroidal anti-inflammatory drugs
PT-INR: Prothrombin time-international normalized ratio
PR: Pulse rate
PRBC: Packed red blood cell
PS: Performance status
RCT: Randomized controlled trial
SRH: Stigmata of recent hemorrhage
SBP: Systolic blood pressure
SD: Standard deviation
UGIB: Upper gastrointestinal bleeding
