# Supplementary material for: Outcomes in high and low volume hospitals in patients with acute hematochezia in a cohort study
Source: Sci Rep. 2021 Oct 13;11:20373. doi: 10.1038/s41598-021-99832-6 (PMC8514573; doi:10.1038/s41598-021-99832-6)
Supplement: Supplementary file 1 — Supplementary Information 1. [file 41598_2021_99832_MOESM1_ESM.docx]

**Supplementary Table 1. Characteristics of hospitals participating in the CODE-BLUE-J study.**

|  | **Hospitals (Approval number in ethics committee)** | **EMS volume in 2019** |
| --- | --- | --- |
| 1 | National Center for Global Health and Medicine (3539) | 12000 |
| 2 | St, Luke’s International Hospital (St. Luke’s International University) (20-R012) | 11000 |
| 3 | Kitano Hospital (P200500400) | 9246 |
| 4 | Nippon Medical School, Graduate School of Medicine (B-2020-147) | 7731 |
| 5 | Tokyo Saiseikai Central Hospital (2020-015-01) | 6391 |
| 6 | Tokyo Metropolitan Bokutoh Hospital (02-024) | 6345 |
| 7 | Saiseikai Yokohamashi Tobu Hospital (20200030) | 6330 |
| 8 | Toranomon Hospital (2021) | 6315 |
| 9 | St Marianna University School of Medicine (4802) | 6200 |
| 10 | Tokyo Medical University (T20190244) | 6000 |
| 11 | The University of Tokyo (2020067NI) | 5859 |
| 12 | Kitasato University, School of Medicine (C20-174) | 5267 |
| 13 | Kagoshima City Hospital (2020-25) | 5225 |
| 14 | Japanese Red Cross Shizuoka Hospital (Jun-20) | 5181 |
| 15 | Tokyo Shinagawa Hospital (20-A-04) | 5075 |
| 16 | Naha City Hospital (2004a4) | 4800 |
| 17 | Hiroshima city asa citizens hospital (2002/1/24) | 4600 |
| 18 | Kawasaki Medical School Hospital (3890) | 4532 |
| 19 | National Hospital Organization Kyoto Medical Center (20-020) | 4455 |
| 20 | Nara City Hospital (NCH倫20-8) | 4298 |
| 21 | University of Tsukuba (R02-030) | 4138 |
| 22 | Iwate Medical University (MH2020-050) | 4051 |
| 23 | Chiba Hokusoh Hospital, Nippon Medical School (802) | 4017 |
| 24 | Kawasaki Medical School General Medical Center (3890) | 3994 |
| 25 | Suita Municipal Hospital (2020-研2) | 3854 |
| 26 | National Hospital Organization Kyushu Medical Center (20C065) | 3500 |
| 27 | Fukuoka University Chikushi Hospital (C20-052) | 3454 |
| 28 | National Hospital Organization Fukuokahigashi Medical Center (2020-臨-2) | 3500 |
| 29 | Saga-Ken Medical Centre Koseikan (20-01-01-03) | 3195 |
| 30 | Fukuoka University Hospital (U20-05-016) | 3454 |
| 31 | National Defense Medical College (4217) | 2840 |
| 32 | Niigata University Medical and Dental Hospital (2020-0052) | 2695 |
| 33 | Kumamoto University Hospital (2040) | 2555 |
| 34 | Fukushima Medical University (一般2020-112) | 2449 |
| 35 | Kagoshima Medical Center (2020-22) | 2263 |
| 36 | Shuto General Hospital (H31-24) | 2234 |
| 37 | Nagoya University Hospital (2020-0152) | 2172 |
| 38 | University of the Ryukyus Hospital (1656) | 2150 |
| 39 | Kagoshima Prefectural Oshima Hospital (97) | 2018 |
| 40 | Graduate School of Medical Sciences, Kyushu University (2020-289) | 1950 |
| 41 | Kirishima City medical association medical center (202005) | 1920 |
| 42 | Akita University Graduate School of Medicine (2491) | 1774 |
| 43 | Izumi General Medical Center (60) | 1762 |
| 44 | Hirosaki University Hospital (2020-32) | 1739 |
| 45 | Kagoshima University Graduate School of Medical and Dental Sciences (200041疫) | 1700 |
| 46 | Oita University Hospital (1845) | 1682 |
| 47 | Tokyo Metropolitan Cancer and Infectious Diseases Center Komagome Hospital (2503) | 1565 |
| 48 | University of Miyazaki Hospital (0-0734) | 1465 |
| 49 | Kagoshima Kouseiren Hospital (215) | 209 |

**Abbreviation.** EMS, emergency medical service
