# Supplementary material for: Outcomes in high and low volume hospitals in patients with acute hematochezia in a cohort study
Source: Sci Rep. 2021 Oct 13;11:20373. doi: 10.1038/s41598-021-99832-6 (PMC8514573; doi:10.1038/s41598-021-99832-6)
Supplement: Supplementary file 2 — Supplementary Information 2. [file 41598_2021_99832_MOESM2_ESM.docx]

**Supplementary data of Stata commands used for path analysis using generalized structural equation modeling (GSEM).**

**Supplementary Figure 1. Association of high-volume hospitals with rebleeding within 30 days without mediators in the matched cohort.**

**Note.** Stata commands used for the analysis: gsem (high_volume -> rebleeding_within_30, family(bernoulli) link(logit)), nocapslatent

**Supplementary Figure 2. Association of high-volume hospitals with rebleeding within 30 days with mediators in the matched cohort.**

**Note.** Stata commands used for the analysis: gsem (high_volume -> rebleeding_within_30, family(bernoulli) link(logit)) (high_volume -> earlycolonoscopy, family(bernoulli) link(logit)) (high_volume -> CT_first, family(bernoulli) link(logit)) (high_volume -> endoscopic_therapies, family(bernoulli) link(logit)) (earlycolonoscopy -> rebleeding_within_30, family(bernoulli) link(logit)) (earlycolonoscopy -> endoscopic_therapies, family(bernoulli) link(logit)) (CT_first -> rebleeding_within_30, family(bernoulli) link(logit)) (CT_first -> earlycolonoscopy, family(bernoulli) link(logit)) (CT_first -> endoscopic_therapies, family(bernoulli) link(logit)) (endoscopic_therapies -> rebleeding_within_30, family(bernoulli) link(logit)), nocapslatent

**Supplementary Figure 3. Association of high-volume hospitals with death within 30 days without mediators in the matched cohort.**

**Note.** Stata commands used for the analysis: gsem (high_volume -> death_within_30, family(bernoulli) link(logit)), nocapslatent

**Supplementary Figure 4. Association of high-volume hospitals with death within 30 days with mediators in the matched cohort.**

**Note.** Stata commands used for the analysis: gsem (high_volume -> death_within_30, family(bernoulli) link(logit)) (high_volume -> earlycolonoscopy, family(bernoulli) link(logit)) (high_volume -> CT_first, family(bernoulli) link(logit)) (high_volume -> endoscopic_therapies, family(bernoulli) link(logit)) (earlycolonoscopy -> death_within_30, family(bernoulli) link(logit)) (earlycolonoscopy -> endoscopic_therapies, family(bernoulli) link(logit)) (CT_first -> death_within_30, family(bernoulli) link(logit)) (CT_first -> earlycolonoscopy, family(bernoulli) link(logit)) (CT_first -> endoscopic_therapies, family(bernoulli) link(logit)) (endoscopic_therapies -> death_within_30, family(bernoulli) link(logit)), nocapslatent
